# Machine learning to predict unintended pregnancy among reproductive-age women in Ethiopia: evidence from EDHS 2016

**DOI:** 10.1186/s12905-024-02893-8

**Published:** 2024-01-23

**Authors:** Daniel Niguse Mamo, Yosef Haile Gebremariam, Jibril Beshir Adem, Shimels Derso Kebede, Agmasie Damtew Walle

**Affiliations:** 1 Department of Health Informatics, School of Public Health, Arbaminch University, Arbaminch, Ethiopia; 2 Department of Public Health, School of Public Health, Arbaminch University, Arbaminch, Ethiopia; 3 Department of Health Informatics, Institute of Public Health, Arsi University, Assela, Ethiopia; 4 Department of Health Informatics, School of Public Health, College of Medicine and Health Science, Wollo University, Dessie, Ethiopia; 5 Department of Health Informatics, college of health science, Mettu University, Mettu, Ethiopia

**Keywords:** Unintended pregnancy, Machine learning, EDHS data, Ethiopia

## Abstract

**Background:**

An unintended pregnancy is a pregnancy that is either unwanted or mistimed, such as when it occurs earlier than desired. It is one of the most important issues the public health system is currently facing, and it comes at a significant cost to society both economically and socially. The burden of an undesired pregnancy still weighs heavily on Ethiopia. The purpose of this study was to assess the effectiveness of machine learning algorithms in predicting unintended pregnancy in Ethiopia and to identify the key predictors.

**Method:**

Machine learning techniques were used in the study to analyze secondary data from the 2016 Ethiopian Demographic and Health Survey. To predict and identify significant determinants of unintended pregnancy using Python software, six machine-learning algorithms were applied to a total sample of 7193 women. The top unplanned pregnancy predictors were chosen using the feature importance technique. The effectiveness of such models was evaluated using sensitivity, specificity, accuracy, and area under the curve.

**Result:**

The ExtraTrees classifier was chosen as the top machine learning model after various performance evaluations. The region, the ideal number of children, religion, wealth index, age at first sex, husband education, refusal sex, total births, age at first birth, and mother’s educational status are identified as contributing factors in that predict unintended pregnancy.

**Conclusion:**

The ExtraTrees machine learning model has a better predictive performance for identifying predictors of unintended pregnancies among the chosen algorithms and could improve with better policy decision-making in this area. Using these important features to help direct appropriate policy can significantly increase the chances of mother survival.

## Background

An unintended pregnancy is a pregnancy that is either unwanted or mistimed, such as when it occurs earlier than desired. It is one of the most important issues the public health system is currently facing, and it comes at a significant cost to society both economically and socially. It results in decreased workforce productivity and quality of life [1, 2]. Between 2015 and 2019, there were 121 million unintended pregnancies worldwide. Every year, 61% of pregnancies result in abortions. Although unintended births have decreased globally, there has been an uneven distribution between high-income and low-income nations. The prevalence rate in high-income countries is 66 per 1000 pregnancies however; it was 93 per 100 women in middle and low-income countries. The burden of an undesired pregnancy still weighs heavily on Ethiopia despite the availability of broad family planning services. According to a systematic review conducted in Ethiopia, the overall prevalence of unintended pregnancy was 28%. Also, results from the 2016 Ethiopian Demographic and Health Survey (EDHS) support this finding, which indicates that 25% of all births in the previous five years and all ongoing pregnancies were unintended [3–5].

Globally, unintended pregnancies have a variety of detrimental effects on mothers and fetuses. One of the most common negative consequences of unintended pregnancy is induced abortion with its complications. Six out of 10 of all unintended pregnancies end in induced abortion. People with unintended pregnancies frequently turn to unsafe abortion when they encounter obstacles to obtaining a safe, quick, inexpensive, geographically accessible, respectful, and non-discriminatory abortion [6, 7]. The child of an unintended pregnancy is more likely to be maltreated, to be born weighing less than 2,500 g, to die within the first year of life, and to lack the resources necessary for healthy growth. It also affects mothers by making the relationship with their spouse more likely to end in divorce, and she may be more likely to experience physical violence herself. The mother and father can experience financial difficulty and fall short of their aspirations for their careers and education [8].

According to different studies, the prevalence of unintended pregnancies was highest among women who were between the ages of 18 and 24 years, had never used family planning methods, had low income (less than 100% of the federal poverty level), had not completed high school, had a birth interval of fewer than two years, was living in rural areas, was pregnant only by their husband’s decision, had gravidity greater than or equal to five, was non-Hispanic black or African American, and was cohabiting but had never married [6–12].

The following recommendations are made to help reduce unintended pregnancies: increasing access to contraception; raising awareness of the importance of feelings, attitudes, and motivation in using contraception and preventing unintended pregnancies; developing and meticulously evaluating a variety of local programs, and encouraging research to create new contraceptives. The new guideline of abortion care also recommends straightforward primary care interventions that involve assuring access to medical abortion pills, ensuring that correct care information is available to all those who need it, improving the quality of abortion care delivered to women and girls, and task-sharing by a wider range of health providers [8, 13].

The potential causes of unintended pregnancy have been the subject of numerous research investigations using traditional statistical analysis methods [6, 14–17]. Nevertheless, no prior studies have attempted to use machine learning to predict unintended pregnancy and identify predictive factors. As a result, when the number of input variables and potential correlations rises, previously employed statistical procedures become less accurate, producing incorrect conclusions [18]. Machine learning was used more effectively [19] and machine learning methods are a good solution to these issues because they can capture complicated and nonlinear correlations in the data, improving prediction accuracy above traditional regression models. So, the purpose of this work was to use the most advanced machine learning models to predict unintended pregnancy and identify its predictors.

## Method

### Data source and population

This study relied on the 2016 Ethiopian Demographic and Health Survey (EDHS), a nationally representative survey that was conducted from January 18 to June 27, 2016. The survey’s sample was divided into two groups and then selected in two stages. A total of 645 EAs were chosen in the first stage, with the chance of selection inversely correlated with the size of the EA (202 in urban regions and 443 in rural areas). 28 households per cluster were chosen in the second stage by a methodical process with an equal probability. A comprehensive amount of data was gathered from 16,650 households, 15,683 female respondents, and 12,688 male respondents on topics such as adult and childhood morbidity and mortality, awareness and attitudes toward HIV/AIDS, and other significant public health issues. These topics included fertility and fertility preference, marriage, awareness and use of family planning methods, as well as issues related to reproductive health [4]. A total weighted sample of 7590 women (15–49 years old) of reproductive age who had birth within the five years before the survey was used.

### Study features

The dependent variable was unintended pregnancy, which encompasses unintended or later-wanted pregnancies. The independent features for this study were the maternal age, maternal occupation, marital status, religion, region parity, household size, wealth index, Husband occupational status, Husband education, residence past miscarriages, knowledge of the ovulation cycle, and distance from the health facility, Ideal number of children, age at first sex, refusal sex, total birth, and age at first birth. To make important independent variables appropriate for analysis, they were recorded or categorized.

### Data processing and analysis

A high-quality dataset is required for machine learning to make predictions. As a result, managing the missing data during the dataset’s pre-processing is an essential step. Encoding data is a fundamental and necessary procedure that is included in data pre-processing. Categorical variables were encoded using one-hot and label encoding. Values that fall into two or more categories and are discrete rather than continuous are said to be categorical. One hot encoding and label encoding technique were used in this work to encode categorical variables [20].

### Data analysis

In this study, descriptive statistics were used to describe the socio-demographic characteristics using frequency and percentage. Data analysis stages included pre-processing the data, feature selection, data splitting, addressing imbalanced data, model building, and model performance testing. Python version 3 was the tool used in this study.

### Feature selection method

The goal of feature selection is to rank and prioritize the most important predictors in the dataset. This is determined by computing the information gain values for each of the selected variables. To find the major factors that significantly result in unintended pregnancy, we used a decision tree classifier, extra trees classifier, XGBoost classifier, gradient boosting classifier, and a random forest model in this work. The higher information gain values indicate significant variables and their class have strong associations. The top ten information values were chosen at random. It is a relatively effective method for reducing model complexity and accelerating the processing of machine learning algorithms [21].

### Data split

For machine learning approaches, the dataset is randomly divided into two parts: one is a training dataset that trains the model, and the second is a test dataset that predicts the response variable and sees if the predicted outcome is similar to the actual outcomes. The validation dataset is also taken into consideration for the parameter estimates to be incorporated into the training models [22]. However, The complete dataset for this study was divided into ten folds using the stratified tenfold cross-validation approach.

### Imbalance data handling

The effectiveness of machine learning algorithms is frequently assessed using predictive accuracy, however, due to the imbalance in the data, it is challenging to identify the root cause of unintended pregnancy. To balance the majority and minority classes in this study, the Synthetic Minority Oversampling Technique (SMOTE) [23] was employed. SMOTE is a pre-processing method for learning algorithms that effectively handles class imbalance by oversampling imbalanced datasets. By linearly overlaying at random between a few samples and their neighbors, it generates a new sample [24].

### Method of building a predictive model

The most effective models were picked to do the training after the data was arranged and split into training and testing samples. To produce a prediction, it was necessary to select the appropriate classifiers for the result variable’s categorical nature, which made the challenge a classification task. In this work, six supervised classification methods were employed. The ExtraTrees classifier, Random Forest, Decision Tree, Logistic Regression, Gradient Boosting, and XGBoost were used for this study. The algorithms were chosen for their accuracy, training time, ability to handle missing data, and ease of understanding and learning.

### Performance evaluation for predictive model

Following model training, each model’s performances are assessed and contrasted with one another. Based on the confusion matrix, the prediction models’ performance was assessed. Precision, sensitivity, specificity, F1-score, and area under the receiver-operating characteristic (AUC-ROC) were utilized in this study to evaluate the model’s performance.

The confusion matrix is a common performance measuring tool used in machine learning classification tasks and is used to describe a model’s output as a binary class [25]. The performance of ML models was also visualized using the ROC curve (or receiver operating characteristic curve) (Table 1).

**Table 1:** Confusion matrix and different derived metrics adapted from [25]

	Predictive positive		Predictive negative
Actual positive	True Positive (TP)	False Negative (FN)	
Actual negative	False Positive (FP)	True Negative (TN)	

According to the confusion matrix above, the following lists recall (sensitivity), (specificity), precision, and accuracy were derived

1 $$\text{R}\text{e}\text{c}\text{a}\text{l}\text{l} \left(\text{S}\text{e}\text{n}\text{s}\text{i}\text{t}\text{i}\text{v}\text{i}\text{t}\text{y}\right) =\frac{\text{T}\text{P}}{\text{T}\text{P}+\text{F}\text{N} }$$

2 $$\text{S}\text{p}\text{e}\text{c}\text{i}\text{f}\text{i}\text{c}\text{i}\text{t}\text{y} =\frac{\text{T}\text{N}}{\text{T}\text{N}+\text{F}\text{P} }$$

3 $$\text{P}\text{r}\text{e}\text{c}\text{i}\text{s}\text{i}\text{o}\text{n} =\frac{\text{T}\text{P}}{\text{T}\text{P}+\text{F}\text{P} }$$

4 $$\text{A}\text{c}\text{c}\text{u}\text{r}\text{a}\text{c}\text{y}=\frac{\text{T}\text{P}+\text{T}\text{N}}{\text{T}\text{N}+\text{T}\text{P}+\text{F}\text{P}+\text{F}\text{N} }$$

5 $$\text{F}1 =2\text{*}\frac{\text{R}\text{e}\text{c}\text{a}\text{l}\text{l}\text{*}\text{P}\text{r}\text{e}\text{c}\text{i}\text{s}\text{i}\text{o}\text{n}}{\text{R}\text{e}\text{c}\text{a}\text{l}\text{l} +\text{P}\text{r}\text{e}\text{c}\text{i}\text{s}\text{i}\text{o}\text{n} }$$

**Fig. 1:**
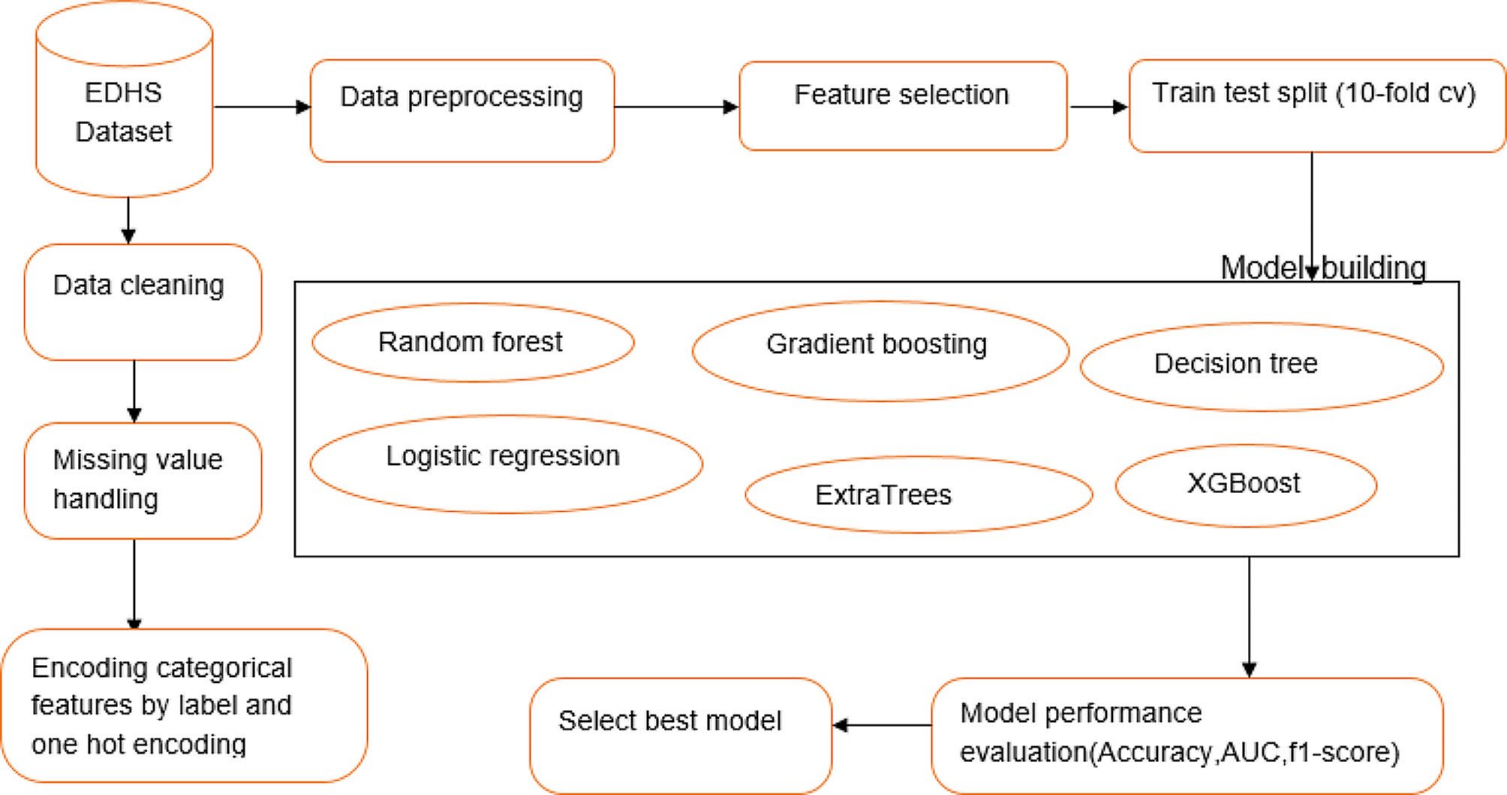
Workflow of machine learning for unintended pregnancy prediction

In summary, Fig. 1 shows the machine learning process used in this study.

## Results

### Sociodemographic characteristics of participants

From the total number of reproductive-age women who had unintended, this study includes 7589 women who were reproductive age. 69.36% (5264) were between the ages of 20 and 34. 92.58% (7026) women were married and 79% (6042) lived in a rural area and 41.24% (3129) lived in Oromia. More than half of the women had not educated, and 33.10% (2512) of all women were orthodox (Table 2).

**Table 2:** Sociodemographic characteristics of reproductive age women in Ethiopia, EDHS 2016, March 2023 (*n* = 7589)

Features	Category	Frequency	Percent (%)
Maternal Age			
	15–19	413	5.44
	20–34	5264	69.36
	35–49	1912	25.20
Residence			
	Urban	1547	20.4
	Rural	6042	79.6
Marital Status			
	Single	68	0.90
	Married	7026	92.58
	Widowed	104	1.37
	Divorced	392	5.16
Maternal educational status			
	No education	4629	61.00
	Primary	2028	26.72
	Secondary	594	7.83
	Higher	338	4.46
Husband education			
	No education	3421	48.84
	Primary	2279	32.42
	Secondary	765	10.89
	Higher	551	7.85
Husband occupational status			
	Not employed	742	10.56
	Employed	6217	88.49
	do not know	67	0.95
Maternal work status			
	Not working	4257	56.09
	Working	3332	43.91
Wealth index			
	Poor	3964	52.23
	Middle	1330	17.52
	Rich	2295	30.24
Religion			
	Orthodox	2512	33.10
	Protestant	1457	19.20
	Muslim	3432	45.22
	Other	189	2.49
Region			
	Addis Abeba	198	2.61
	Afar	71	0.94
	Amhara	1,632	21.51
	Benishangul	81	1.06
	Dire Dawa	33	0.44
	Gambela	21	0.27
	Harari	17	0.23
	Oromia	3129	41.24
	SNNPR	1601	21.09
	Somali	269	3.54
	Tigray	537	7.07

### Imbalance data handling

Unbalanced data handling was a key strategy for this study to handle the problem of unbalanced data and boost the performance of the machine learning algorithms. An imbalanced dataset was balanced using the SMOTE sampling method, and the accuracy and AUC based on the chosen machine learning algorithms were compared for the balanced and unbalanced datasets. When compared to another classifier in the unbalanced dataset, gradient boosting performed better with an AUC of 0.682, while logistic regression had a higher AUC of 0.668. The Extra tree classifier in the SMOTE has a higher accuracy of 84.93% and an AUC of 0.926. Moreover, the Random forest also outperformed next to the Extratrees classifier on a balanced dataset, with the test accuracy and AUC values of 84.40 and 0.924, respectively (Table 3).

**Table 3:** Compares imbalanced data handling techniques using accuracy and Area under the curve (AUC)

Algorithms	Comparison method	Unbalanced	SMOTE
Logistic Regression	Accuracy (%)	80.25	70.00
	AUC	**0.668**	0.775
Decision Tree	Accuracy (%)	66.75	75.95
	AUC	0.557	0.760
Random Forest	Accuracy (%)	**79.41**	**84.40**
	AUC	0.659	**0.924**
Gradient Boosting	Accuracy (%)	79.13	74.91
	AUC	0.682	0.824
XGBoost	Accuracy (%)	77.32	82.22
	AUC	0.641	0.898
Extra Tree classifier	Accuracy (%)	78.74	84.93
	AUC	0.628	0.926

**SMOTE**: Synthetic Minority Over-sampling Technique, **AUC**: Area Under Curve, **Underline** and **bold** numbers were the highest score of the classifier

**Fig. 2:**
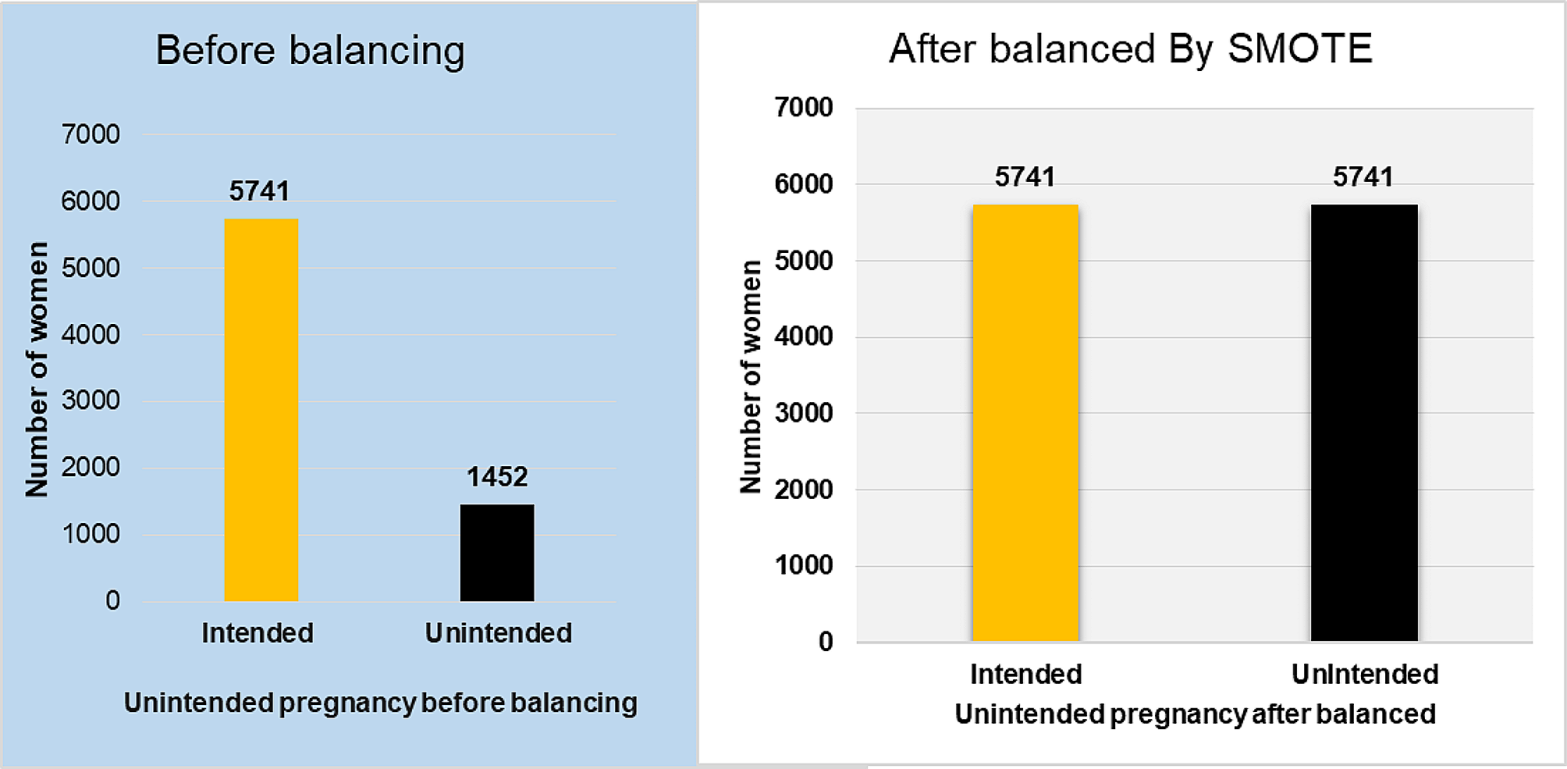
Before unbalanced and after balancing the target feature

Machine learning is difficult with unbalanced data because values from the minority class or rarely occurring classes are wrongly categorized as instances of the majority class, which lowers the performance of the classifying algorithm. After all, the classifier is overwhelmed by the dominant class and ignores the unintended class, which is the minority class. After SMOTE was applied to the unbalanced dataset, the overall number of records rose. (Fig. 2). We mainly used AUC to compare the classifier and balanced sampling method.

### Implementation of unintended pregnancy prediction models

**Fig. 3:**
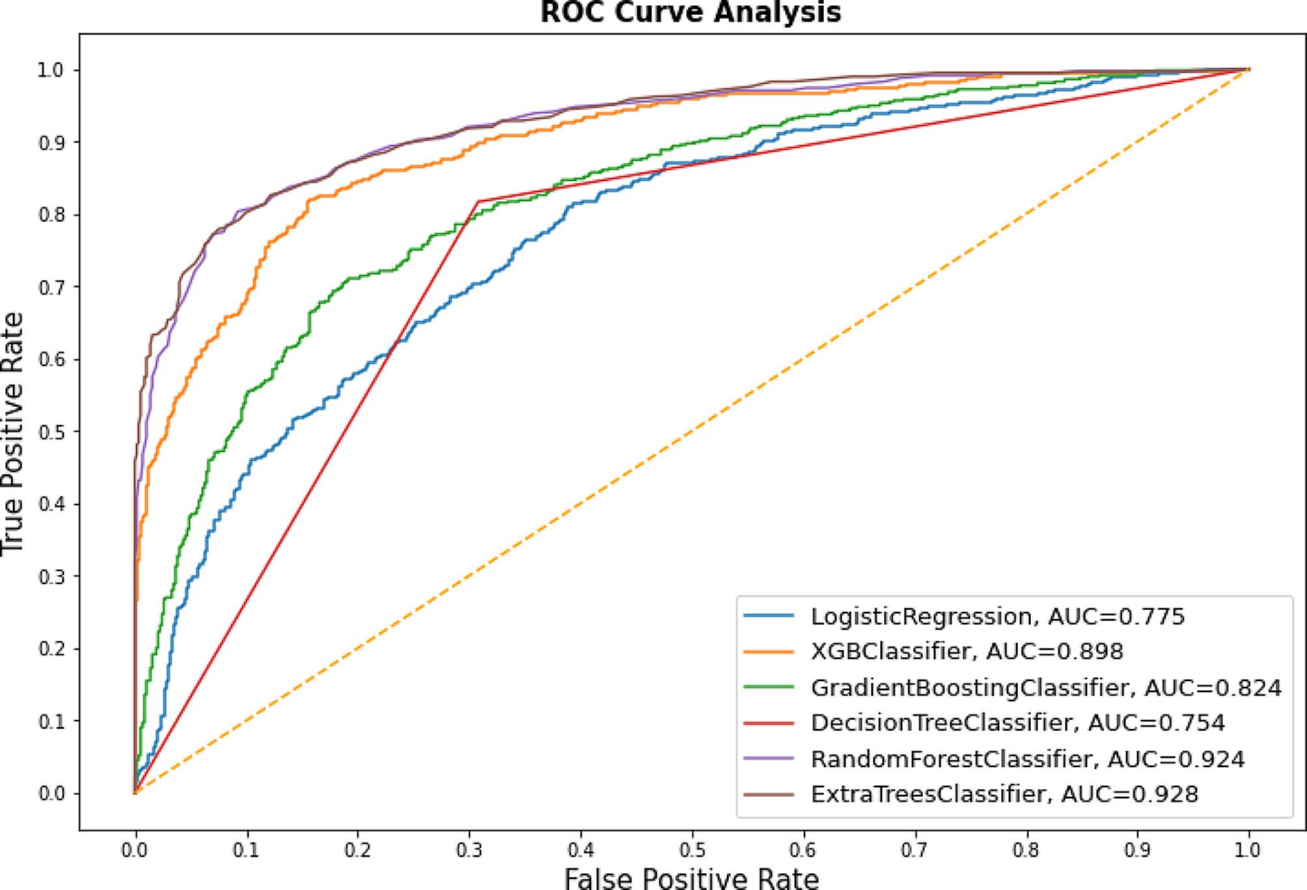
ROC curve shows a balanced dataset using SMOTE

In this study, the data were split into training and test sets, which together made up 90% and 10% of the total data. The model performance, including prediction evaluation metrics, can be evaluated in comparison to various machine learning classifiers. To avoid overfitting, the popular 10-fold cross-validation method was applied to this study. The experiments were mainly divided into two sections: the first section trained the different classification algorithms using 32 features from an imbalanced dataset, and the second section employed a balanced sampling strategy to determine which model with 32 features was the best. High accuracy, precision, sensitivity, specificity, f1-score, and AUC were obtained by applying various machine learning classification algorithms like Logistic regression, decision tree, random forest, gradient boosting, XGBoost, and ExtraTrees) to the balanced data using SMOTE. In comparison to other algorithms, ExtraTrees produces better accuracy and results in performance metrics. The ExtraTrees classifier (AUC = 0.928) outperforms all other classifiers in terms of performance metrics and is the best in foretelling unintended pregnancies, as shown by the ROC curve in Fig. 3. Alongside the ExtraTrees classifier, the performance of random forest (AUC = 0.924), XGBoost (0.898), logistic regression (AUC = 0.775), XGBoost (0.898), gradient boosting classifier (AUC = 0.824), and decision tree classifier (AUC = 0.76) was also impressive (Fig. 3).

### Extratrees classifier performance

From the balanced dataset, the ExtraTrees classifier’s performance was quite strong compared to other selected classifiers. The hyper-parameter tuning and feature selection were carried out after the best model had been chosen. The important predictor of unintended pregnancy was established to compare the model’s performance.

### Tuning an ExtraTrees classifier with grid serach CV

**Fig. 4:**
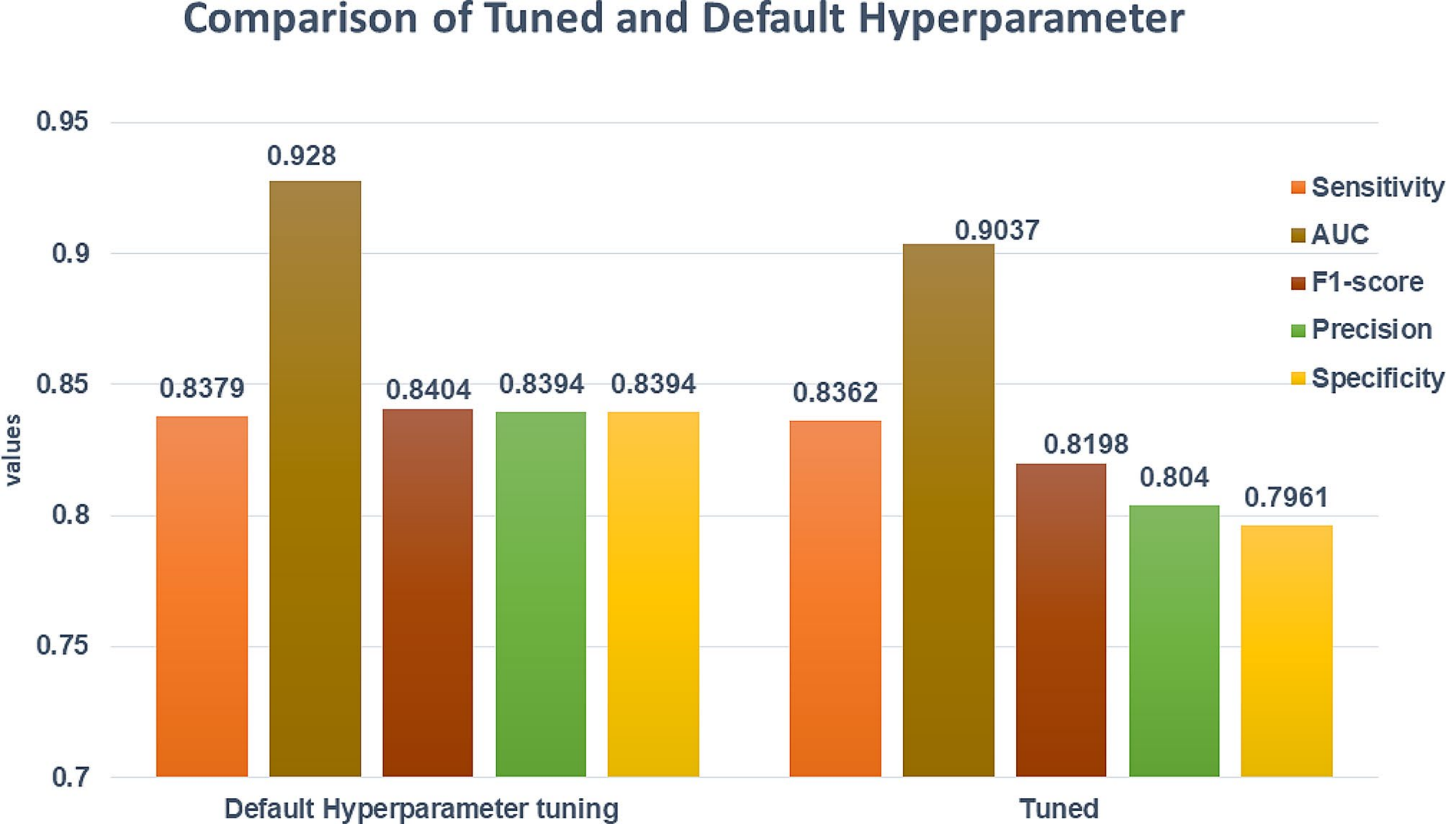
Comparison of tuned and default hyperparameter using ExtraTrees classifier

After selecting the best model, this study applied the hyperparameter tuning to compare it with the default hyperparameter tuning. Figure 4 shows that default hyperparameter tuning was higher performed than hyperparameter tuning using the best classifier of the ExtraTrees Model. According to the results, the ExtraTrees Model classifier with tuned hyperparameters was less performed than the ExtraTrees classifier with the default hyperparameter. Therefore, this study used the ExtraTrees classifier with a default hyperparameter with the tuning of sensitivity, specificity, precision, and f1-score of 83.79%, 83.94%, 83.94%, and 84.04%, respectively. The ExtraTrees Model classifier with default hyperparameter tuning had the highest AUC value, which means that the classifier properly identified unintended or unplanned. Then this study applied the default hyperparameter tuning (Fig. 4).

### Top features from the chosen classifier

This experiment was performed to examine the classifier’s ability to predict unintended pregnancy and the impact of feature selection. Based on all chosen classifiers, this study identified the features that predict unintended pregnancy to determine which features were the best predictors. The cumulative result of the classifier feature importance was chosen as the suitable way to identify the features that most reliably predict unintended pregnancy for this study using these findings as a guide. The region, the Ideal number of children, religion, wealth index, age at first sex, husband education, refusal sex, total birth, age at first birth, and Mother’s Educational Status were the factors that had the greatest impact on unintended pregnancy out of all the predictors. Table 4 shows that from the chosen classifier, the top ten features were selected using the median results (Table 4).

**Table 4:** Compares selected machine learning models in choosing the top features

R.No	Top Features	ETC	GB	RF	XGB	DT	LR	Median
**1**	Region	0.0727	0.1047	0.0212	0.0336	0.1181	0.0308	0.0727
**2**	Ideal number Children	0.0601	0.0613	0.0495	0.1558	0.0557	0.5931	0.0601
**3**	Wealth index	0.0459	0.0461	0.0318	0.0472	0.0469	0.3090	0.0461
**4**	Husband education	0.0423	0.0461	0.0220	0.0107	0.0478	0.0279	0.0423
**5**	Religion	0.0468	0.0476	0.0218	0.0162	0.0418	0.1262	0.0418
**6**	Age at first sex	0.0417	0.0391	0.0271	0.0488	0.0411	0.3169	0.0411
**7**	Total birth	0.0378	0.0363	0.0246	0.0155	0.0418	0.0497	0.0364
**8**	Refuse sex	0.0379	0.0364	0.0194	0.0010	0.0344	0.0756	0.0344
**9**	Age at 1st birth	0.0344	0.0329	0.0330	0.0666	0.0362	0.3659	0.0344
**10**	Maternal Educational status	0.0335	0.0337	0.0231	0.0149	0.0373	0.1667	0.0336

**RF**: Random Forest; ExtraTrees; **DT**: Decision Tree; **LR**: Logistic Regression; **GB**: Gradient Boost; **XGBoost**: Extreme Gradient Boosting

### ExtraTrees classifier features importance

**Fig. 5:**
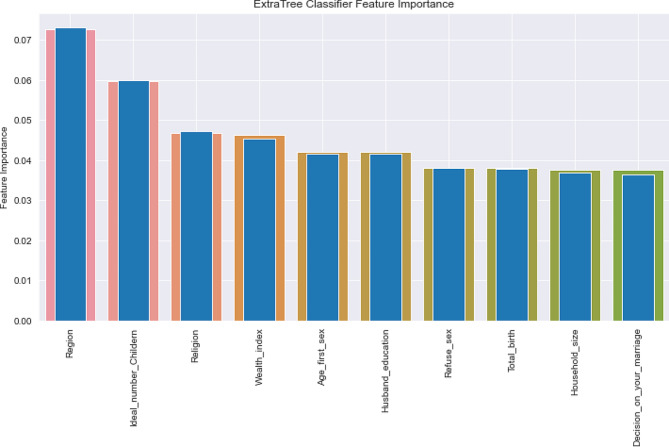
Relevant features selected by an ExtraTrees feature importance

Relevant features selected by an ExtraTrees classifier show that at the bottom were identified as the top predictors of unintended pregnancy. Of all features, region, ideal number of children, religion wealth index, age at first sex, husband education, household size, refusal sex, total birth, and decision on marriage were top predictors (Fig. 5).

## Discussion

According to earlier research on this topic, Ethiopia has one of the highest rates of unintended pregnancies worldwide [26–29]. Findings also revealed that while the prevalence of unwanted pregnancies has occasionally declined in the nation, more work is still needed to support this pattern and manage the phenomenon’s undesirable repercussions. Machine learning models are regarded as state-of-the-art approaches and techniques for quick and accurate problem-solving. This study has aimed to predict and identify the predictors of unintended pregnancy and build the best performance of a machine learning classifier. Six machine learning algorithms such as Logistic Regression, Decision Tree, Random Forest, Gradient Boosting, XGBoost, and ExtraTrees, were applied to predict unintended pregnancy in Ethiopia using EDHS 2016 data.

The above models were chosen to build and evaluate the best predictive model using the key predictors, which will increase model prediction accuracy and generalizability. stratified 10-fold cross-validation has been used to train the classifiers on a set of training data. To determine the optimal accuracy, several tests were conducted applying both balanced and unbalanced datasets. The outcome demonstrated that imbalanced data produced low-performance metrics. To balance the unbalanced data, this study used the SMOTE balancing sampling approach. AUC, recall, precision, and accuracy performance evaluations showed that the ExtraTrees classifier performed better than all other selected classifiers (84.75%, 84.66%, 84.81%, and (0.925)., respectively). As a result, this classifier was selected in our study for the prediction of unintended pregnancy.

Using the relevance values of independent features for the ExtraTrees classifier, this study identified the key influencing factors for unintended pregnancy. The most significant variables that contribute to higher performance in unintended pregnancy prediction were found using the average results of all classifiers used in the feature selection process. The important predictors of unintended pregnancy among all independent characteristics included region, the ideal number of children, religion, wealth index, age at first sex, husband education, refusal sex, total birth, age at first birth, and mother’s educational status.

The region was a very important predictor of unintended pregnancy. This research supports a systematic review and meta-analysis of an Ethiopian observational study [6], as well as a study using EDHS 2016 data that found that different regions of Ethiopia [14] had higher rates of unintended pregnancies. The sociodemographic variations between the individuals in each region could be one of the causes.

The machine learning classifier identified that the wealth index was a highly important feature for predicting unintended pregnancies. This finding is supported by research conducted in India [30], Bangladesh [31], Iran [32], Nepal [33], Nigeria [34], Kenya [35], and across different parts of Ethiopia [36–38], revealed that women who have high wealth status are more empowered to take charge of their sexual and reproductive health matters than women who have poorer wealth status. The relationship between income status, occupation, and unintended pregnancies may be explained by the connection between formal employment and social networks, and earning potential [39].

Religion was an important feature in predicting unintended pregnancy. previous studies conducted in Bangladesh [31], Nepal [40], Addis Zemen, and Ethiopia [41], and a study using EDHS 2016 data in Ethiopia [14] revealed that Women who had a religion tend to be highly associated with unintended pregnancy. The possible explanations for the association include the fact that women believe that every child is a gift from God and that their religion discourages the use of contraception. Thus, mothers who follow a particular faith do not think that unintended pregnancy occurs.

Based on the finding of this study, the husband’s education and the mother’s educational status of the respondent were found other relevant features for predicting unintended pregnancy. This finding is supported by research conducted in Russia [42], Bangladesh [43], Uganda [44], Malawi [45], and Southern Ethiopia [46], which reported that husbands and mothers who had educational status were more likely associated with unintended pregnancy. The possible explanation might be due.

Other relevant features of unintended pregnancy were the Ideal number of children, age at first sex, refusal of sex, total birth, and age at first birth.

In findings, our study shows that machine learning techniques can be used to identify predictive characteristics related to unwanted pregnancy. Machine learning methods appear to be useful for determining which indicators are most important for predicting an unplanned pregnancy. Our study model might help with the crucial public health problem of identifying and managing unintended pregnancies.

For predicting unintended or unplanned pregnancies, the suggested method has the best ROC, accuracy, precision, recall, and specificity. This prediction is in support of providing women with comprehensive services and extended working hours. Effective predictive modeling may raise medical care standards and increase maternal survival. Therefore, the prediction models of unintended pregnancy developed in our work can significantly contribute by detecting women with undesired or unintended pregnancies and adopting the most effective supportive measures, such as offering training or any other form of information transmission. This might reduce misunderstanding by providing quantitative, unbiased, and research-based models for risk classification, prediction, and ultimately care planning. This work cannot be considered complete without its limitations. In contrast to the statistical model, the machine learning model’s result lacks a coefficient and odds ratio, making it challenging to determine how much and in which direction various factors impact the final result. In addition, Machine algorithms are primarily less interpretable because they lack parameters and typically identify or anticipate particular variables according to how significant a part they played in the current study’s determination of the unwanted pregnancy.

## Conclusion

In predicting unintended pregnancy factors in Ethiopia, the ExtraTrees classifier has a somewhat higher predictive ability than other selected machine learning classifiers. By using the ExtraTrees classifier to choose the desired features related to unintended pregnancy, we found that region, the ideal number of children, religion, wealth index, age at first sex, husband education, refusal sex, total birth, age at first birth, and mother educational status were the significant predictors of unintended pregnancy. This work emphasizes the use of machine learning algorithms to predict and better comprehend top significant unintended pregnancy predictor variables to improve essential policy directions.

## Data Availability

The corresponding author will provide the datasets used in the current work upon reasonable request.

## Acronyms and abbreviations

AUC: Area Under Curve
CSV: Comma Separated Values
EDHS: Ethiopia Demographic and Health Survey
ML: Machine Learning
ROC: Receiver Operating Characteristic
SMOTE: Synthetic Minority Over-sampling Technique
SNNP: South Nation Nationality People
WHO: World Health Organization
XGBoost: EXtreme Gradient Boosting

## References

[1] Control Cod. Reproductive health: Unintended Pregnancy. 2022. Accessed on 10/3/2023. Available from: https://www.cdc.gov/reproductivehealth/contraception/unintendedpregnancy/index.htm.

[2] Organisation WH. Fat sheet: Abortion 2021. Accessed on 10/3/2023. Available from: https://www.who.int/news-room/fact-sheets/detail/abortion.

[3] Bearak J, Popinchalk A, Ganatra B, Moller A-B, Tunçalp Ö, Beavin C (2020). Unintended pregnancy and abortion by income, region, and the legal status of abortion: estimates from a comprehensive model for 1990–2019. The Lancet Global Health.

[4] Central Statistical Agency - CSA/, Ethiopia ICF. Ethiopia Demographic and Health Survey 2016. Addis Ababa, Ethiopia: CSA and ICF; 2017. Available from: http://dhsprogram.com/pubs/pdf/FR328/FR328.pdf.

[5] Nikonovas T, Spessa A, Doerr SH, Clay GD, Mezbahuddin S (2020). Near-complete loss of fire-resistant primary tropical forest cover in Sumatra and Kalimantan. Commun Earth Environ.

[6] Alene M, Yismaw L, Berelie Y, Kassie B, Yeshambel R, Assemie MA (2020). Prevalence and determinants of unintended pregnancy in Ethiopia: a systematic review and meta-analysis of observational studies. PLoS ONE.

[7] Eseta WA, Lemma TD, Geta ET. Magnitude and determinants of dropout from community-based health insurance among households in manna district, Jimma zone, Southwest Ethiopia. *ClinicoEconomics and Outcomes Research*. 2020:747 – 60. 10.2147/CEOR.S284702 PMC7751608 33364800

[8] Institute of Medicine Committee on Unintended P. In: Brown SS, Eisenberg L, editors. The Best Intentions: Unintended Pregnancy and the Well-Being of Children and Families. Washington (DC): National Academies Press (US) Copyright 1995 by the National Academy of Sciences. All rights reserved.; 1995. 25121228

[9] Nigussie K, Degu G, Chanie H, Edemealem H. Magnitude of unintended pregnancy and associated factors among pregnant women in Debre Markos Town, East Gojjam Zone, Northwest Ethiopia: a cross-sectional study. Int J women’s health. 2021:129–39. 10.2147/IJWH.S275346 PMC7851705 33542661

[10] Barrow A, Jobe A, Barrow S, Touray E, Ekholuenetale M (2022). Prevalence and factors associated with unplanned pregnancy in the Gambia: findings from 2018 population-based survey. BMC Pregnancy Childbirth.

[11] Bekele H, Dheressa M, Mengistie B, Sintayehu Y, Fekadu G (2020). Unintended pregnancy and Associated Factors among pregnant women attending Antenatal Care at Bako Tibe District Public Health Facility, Oromia Region, Ethiopia. J Pregnancy.

[12] Tenaw SG, Chemir F, Zewudie BT, Chekole B, Argaw M, Mesfin Y et al. Unintended pregnancy and associated factors among women attending antenatal care in public hospitals during COVID-19 pandemic, Southwest Ethiopia: a cross-sectional study. Open Access Journal of Contraception. 2022:9–16. 10.2147/OAJC.S350467 PMC8784268 35082537

[13] Organization WH. Abortion care guideline. 2022. Available from: https://www.who.int/news-room/fact-sheets/detail/abortion. 35344310

[14] Teshale AB, Tesema GA (2020). Magnitude and associated factors of unintended pregnancy in Ethiopia: a multilevel analysis using 2016 EDHS data. BMC Pregnancy Childbirth.

[15] Fite RO, Mohammedamin A, Abebe TW (2018). Unintended pregnancy and associated factors among pregnant women in Arsi Negele Woreda, West Arsi Zone, Ethiopia. BMC Res Notes.

[16] Zeleke LB, Alemu AA, Kassahun EA, Aynalem BY, Hassen HY, Kassa GM (2021). Individual and community level factors associated with unintended pregnancy among pregnant women in Ethiopia. Sci Rep.

[17] Abame DE, Abera M, Tesfay A, Yohannes Y, Ermias D, Markos T (2019). Relationship between unintended pregnancy and antenatal care use during pregnancy in Hadiya Zone, Southern Ethiopia. J Reprod Infertility.

[18] Chen JH, Asch SM (2017). Machine learning and prediction in medicine—beyond the peak of inflated expectations. N Engl J Med.

[19] Bzdok D, Altman N, Krzywinski M (2018). Statistics versus machine learning. Nat Methods.

[20] Al-Shehari T, Alsowail RA (2021). An insider data leakage detection using one-hot encoding, synthetic minority oversampling and machine learning techniques. Entropy.

[21] Saxena A, Ganguly A, Shrivastava AK. Predicting Chronic Kidney Disease Risk Using Recursive Feature Elimination and Machine Learning.

[22] Géron A. Hands-on machine learning with Scikit-Learn, Keras, and TensorFlow. O’Reilly Media, Inc.; 2022.

[23] Chawla NV, Bowyer KW, Hall LO, Kegelmeyer WP (2002). SMOTE: synthetic minority over-sampling technique. J Artif Intell Res.

[24] Zhou R, Yin W, Li W, Wang Y, Lu J, Li Z et al. Prediction Model for Infectious Disease Health Literacy Based on Synthetic Minority Oversampling Technique Algorithm. *Computational and Mathematical Methods in Medicine*. 2022;2022. 10.1155/2022/8498159 PMC8975663 35371281

[25] Kuhn M, Johnson K. Applied predictive modeling: Springer; 2013.

[26] Kebede KM, Belay AS, Shetano AA (2021). Prevalence and determinants of unintended pregnancy in Ethiopia: narrative synthesis and meta-analysis. Heliyon.

[27] Geda NR, Lako TK (2011). A population-based study on unintended pregnancy among married women in a district in Southern Ethiopia. J Geogr Reg Plann.

[28] Gebremariam Weldearegawi G, Tekola KB, Fseha Teklehaymanot B. Magnitude and associated factors of unintended pregnancy among pregnant women at Saesie Tsaeda Emba Woreda Eastern Zone of Tigray, North Ethiopia, 2018. *Journal of Pregnancy*. 2019;2019. 10.1155/2019/1694808 PMC7029673 32089883

[29] Bekele H, Theresa M, Mengistie B, Sintayehu Y, Fekadu G (2020). Unintended pregnancy and associated factors among pregnant women attending antenatal care at Bako Tibe District Public Health Facility, Oromia Region, Ethiopia. J Pregnancy.

[30] Ram R, Kumar M, Kumari N (2022). Association between women’s autonomy and unintended pregnancy in India. Clin Epidemiol Global Health.

[31] Sarder A, Islam SMS, Talukder A, Ahammed B (2021). Prevalence of unintended pregnancy and its associated factors: evidence from six south Asian countries. PLoS ONE.

[32] Omani-Samani R, Amini Rarani M, Sepidarkish M, Khedmati Morasae E, Maroufizadeh S, Almasi-Hashiani A (2018). Socioeconomic inequality of unintended pregnancy in the Iranian population: a decomposition approach. BMC Public Health.

[33] Bastola K, Neupane S, Hadkhale K, Kinnunen T (2015). Unintended pregnancy among married pregnant women in Nepal. J Womens Health Issues Care.

[34] Izugbara C (2013). Household characteristics and unintended pregnancy among ever-married women in Nigeria. Social Med.

[35] Ikamari L, Izugbara C, Ochako R (2013). Prevalence and determinants of unintended pregnancy among women in Nairobi, Kenya. BMC Pregnancy Childbirth.

[36] Geda YF. Determinants of teenage pregnancy in Ethiopia: a case–control study, 2019. *Current medical issues*. 2019;17(4):112.

[37] Wolde TS, Mekebo GG (2021). Unintended pregnancy and associated factors among pregnant women in rural Ethiopia. JPRI.

[38] Kassie T, Moges G, Ali A, Tefera W (2017). Magnitude and factors associated with unintended pregnancy among pregnant women in Addis Ababa, Ethiopia. Glob J Med PUBLIC Heal.

[39] Ahinkorah BO, Seidu A-A, Appiah F, Oduro JK, Sambah F, Baatiema L (2020). Effect of sexual violence on planned, mistimed and unwanted pregnancies among women of reproductive age in sub-saharan Africa: a multi-country analysis of demographic and health surveys. SSM-population Health.

[40] Adhikari R, Soonthorndhada K, Prasartkul P (2009). Correlates of unintended pregnancy among currently pregnant married women in Nepal. BMC Int Health Hum Rights.

[41] Goshu YA, Yitayew AE (2019). Prevalence and determinant factors of unintended pregnancy among pregnant women attending antenatal clinics of Addis Zemen hospital. PLoS ONE.

[42] Panova O, Kulikov A, Berchtold A, Suris J (2016). Factors associated with unwanted pregnancy among adolescents in Russia. J Pediatr Adolesc Gynecol.

[43] Bishwajit G, Tang S, Yaya S, Feng Z (2017). Unmet need for contraception and its association with unintended pregnancy in Bangladesh. BMC Pregnancy Childbirth.

[44] Wasswa R, Kabagenyi A, Atuhaire L (2020). Determinants of unintended pregnancies among currently married women in Uganda. J Health Popul Nutr.

[45] Hall JA, Barrett G, Phiri T, Copas A, Malata A, Stephenson J (2016). Prevalence and determinants of unintended pregnancy in Mchinji District, Malawi; using a conceptual hierarchy to inform analysis. PLoS ONE.

[46] Seifu CN, Fahey PP, Hailemariam TG, Atlantis E (2020). Association of husbands’ education status with unintended pregnancy in their wives in southern Ethiopia: a cross-sectional study. PLoS ONE.

